# Tolerance to Drought, Low pH and Al Combined Stress in Tibetan Wild Barley Is Associated with Improvement of ATPase and Modulation of Antioxidant Defense System

**DOI:** 10.3390/ijms19113553

**Published:** 2018-11-11

**Authors:** Imrul Mosaddek Ahmed, Umme Aktari Nadira, Cheng-Wei Qiu, Fangbin Cao, Guoping Zhang, Paul Holford, Feibo Wu

**Affiliations:** 1 Institute of Crop Science, Department of Agronomy, College of Agriculture and Biotechnology, Zijingang Campus, Zhejiang University, Hangzhou 310058, China; imrulbau@gmail.com (I.M.A.); kbdnadira@yahoo.com (U.A.N.); 3130100260@zju.edu.cn (C.-W.Q.); fangbincao@163.com (F.C.); zhanggp@zju.edu.cn (G.Z.); 2 Jiangsu Co-Innovation Center for Modern Production Technology of Grain Crops, Yangzhou University, Yangzhou 225009, China; 3 Plant Physiology Division, Bangladesh Agricultural Research Institute, Gazipur-1701, Bangladesh; 4 School of Science and Health, Western Sydney University, Penrith, NSW 2751, Australia; p.holford@uws.edu.au

**Keywords:** aluminum, adenosinetriphosphatase, ATPase, methylglyoxal, polyethyleneglycol, PEG, Tibetan wild barley, *Hordeum vulgare* L. ssp. *spontaneum*, ROS

## Abstract

Aluminum (Al) toxicity and drought are two major constraints on plant growth in acidic soils, negatively affecting crop performance and yield. Genotypic differences in the effects of Al/low pH and polyethyleneglycol (PEG) induced drought stress, applied either individually or in combination, were studied in Tibetan wild (XZ5, drought-tolerant; XZ29, Al-tolerant) and cultivated barley (Al-tolerant Dayton; drought-tolerant Tadmor). Tibetan wild barley XZ5 and XZ29 had significantly higher H^+^-ATPase, Ca^2+^Mg^2+^-ATPase, and Na^+^K^+^-ATPase activities at pH 4.0+Al+PEG than Dayton and Tadmor. Moreover, XZ5 and XZ29 possessed increased levels in reduced ascorbate and glutathione under these conditions, and antioxidant enzyme activities were largely stimulated by exposure to pH 4.0+PEG, pH 4.0+Al, and pH 4.0+Al+PEG, compared to a control and to Dayton and Tadmor. The activity of methylglyoxal (MG) was negatively correlated with increased levels of glyoxalase (Gly) I and Gly II in wild barley. Microscopic imaging of each genotype revealed DNA damage and obvious ultrastructural alterations in leaf cells treated with drought or Al alone, and combined pH 4.0+Al+PEG stress; however, XZ29 and XZ5 were less affected than Dayton and Tadmor. Collectively, the authors findings indicated that the higher tolerance of the wild barley to combined pH 4.0+Al+PEG stress is associated with improved ATPase activities, increased glyoxalase activities, reduced MG, and lower reactive oxygen species levels (like O_2_^−^ and H_2_O_2_) due to increased antioxidant enzyme activities. These results offer a broad comprehension of the mechanisms implicated in barley’s tolerance to the combined stress of Al/low pH and drought, and may provide novel insights into the potential utilization of genetic resources, thereby facilitating the development of barley varieties tolerant to drought and Al/low pH stress.

## 1. Introduction

A great threat to agricultural production is the fact that approximately 40% of arable land worldwide currently faces yield reductions because of cyclical or unpredictable drought [1]. Variations in rainfall distribution and longer dry spells due to global climate change in much of the tropics during the main growing period of crops are increasingly becoming important yield-limiting factors. Aluminum (Al) is one of the most abundant elements in the Earth’s crust, and Al^3+^can be toxic to plants at soil concentrations greater than 2–3 mg/L and at soil pH values of <5.5 [2]. As a result, crop genotypes tolerant to both drought and Al toxicity need to be developed. Therefore, a combination of research approaches is urgently needed in order to understand the nature of multiple stress responses and to create avenues for developing plants that are resistant to multiple stresses yet maintain high yields [3].

The primary effect of Al toxicity is a reduction in root growth, which can severely affect water and nutrient uptake, thereby reducing crop yield [4,5]. It has been reported that Al toxicity produces disturbances in peripheral tissues in root and root thickening of *Vaccinium corymbosum* [6]. Little information has been reported on the effects of Al on shoots. However, Konarska [7] showed that Al exposure reduced the size and thickness of leaf blades, due to a decrease in cell size, as well as an increase in stomata number in the abaxial epidermis, with a simultaneous reduction in their size. These effects were accompanied by a decrease of photosynthetic pigment levels and photosynthetic parameters. Similarly, in cotton plants growing under Al toxicity, a reduction in leaf area and chlorophyll content was found [8]. Likewise, several reports indicate that Al also affects the photosynthetic performance by reducing stomatal conductance and the rate of electron transport [9,10]. In addition, Al induces an accumulation in reactive oxygen species (ROS), leading to oxidative stress in organelles and eventually in cell death [11,12,13].

In low pH soils, chemical constraints and interactions among chemicals limit plant growth. For example, in low pH soils (ranging from 4.5 to 5.5), Al and magnesium are at toxic concentrations and phosphorus, nitrogen, potassium, and calcium deficiencies occur in addition to toxic concentrations of hydrogen ions, all of which affect plant growth [14].

Drought stress can cause several seriously deleterious effects on plant metabolic processes, including water relations, nutrient uptake and metabolism, and photosynthetic assimilation [15]. It was assumed that drought in an acidic Al-toxic soil would aggravate Al toxicity, which would further inhibit root growth and restrict water acquisition from the subsoil and the ability of plants to antagonize drought stress [16]. Studies have revealed that the physiological and biochemical responses of plants to the interaction of drought and salinity are unique and cannot be directly extrapolated from the responses to the two stresses individually [17]. However, little information is available regarding the combined stresses of Al and drought. Consequently, studies on Al and drought combined stresses are of great ecological significance and are required to improve plant tolerance to the combined abiotic stress.

Barley (*Hordeum vulgare* L.) is an important grain and ranks fourth in both production and in area of cultivation of cereal crops in the world [18]. In China, according to the Ministry of Water Resources’ survey, over 25.67 million ha of farmland were affected annually by drought stress during the 15th five-year plan, which caused a reduction in production of 3.5×10^10^ kg and an economic loss of more than 230 billion Chinese yuan. Barley is also one of the most Al-sensitive species among cereals [19]. Acidic soil is distributed across 15 provinces in China, which covers an area of 20.3 million ha and occupies almost 21% of land. Therefore, it is important to identify genetic resources with a potential for tolerance to these different stresses, in order to breed tolerant barley cultivars. Wild barley offers a great source of useful genes and genetic variation for crop improvement [20]. Annual wild barley from the Qinghai–Tibet Plateau is regarded as one of the progenitors of cultivated barley and is rich in genetic diversity [21]. The authors’ previous study reported that Tibetan wild barley was tolerant of the combined stress of drought and Al, which was associated with higher increases in Abscisic acid and callose content, and less Al, zeatinriboside, and ethylene accumulation in roots [22]. However, the underlying physiological, biochemical, and cellular mechanisms involved in Al and drought tolerance in the leaves remain unclear. Therefore, changes in the leaf antioxidant defense system, oxidative injury, DNA damage, and cell ultrastructure under Al, low pH and simulated drought stress using polyethylene glycol (PEG) were investigated to study the Al and PEG tolerance mechanisms in Tibetan wild barley. The authors’ hypothesized that under Al, low pH and PEG-induced drought stress the leaf antioxidant defense system could scavenge excessive ROS and sufficiently protect itself from free radical injury.

## 2. Results

### 2.1. Plant Height, Biomass Accumulation, and Al Concentration

There was significant phenotypic variation in response to pH, PEG-induced drought and Al stress (Figure S1), but the time of appearance and severity of stress symptoms differed among the genotypes. The two wild genotypes (XZ29 and XZ5) were less affected in terms of wilting and the development of yellow necrotic patches, whereas Dayton and Tadmor were affected by PEG treatment and by the combined Al, PEG, and pH treatment.

The effects of PEG and Al, both alone and in combination, on plant height, dry weight, and Al concentration of two barley cultivars are shown in Figure S2. Barley plants treated with low pH (4.0) showed reduced growth (Figure S2a); however, no significant effect was found in dry weight compared to pH 6.0 (control) (Figure S2b). PEG stress significantly decreased plant height and dry weight relative to the control. Plant height and dry weight of all four genotypes were further reduced due to the addition of Al compared to those at pH 4.0 without Al. Plants treated with pH 4.0+Al+PEG showed the most severe reduction in plant height and dry weight. Among the genotypes, maximum plant height and dry weight were observed for XZ29 and XZ5 in the pH 4.0+Al+PEG treatment (Figure S2a,b).

Only aluminum stress significantly increased Al concentration in leaves of the four genotypes; Tadmor was the most affected. Remarkably, lower Al concentration in leaves was found when plants were exposed to pH 4.0+Al+PEG compared with plants treated with A1 alone, and a significant genotypic difference was observed. The Al-tolerant genotype, XZ29, had a lower Al content than the other three genotypes (Figure S2c).

### 2.2. Photosynthetic Parameters

The photosynthetic parameters of the four genotypes are shown in Figure S3. Barley plants exposed to low pH (4.0) showed significant decreases in net photosynthetic rate (Pn), stomatal conductance (Gs), and transpiration rate (Tr) in the leaves of all four genotypes (Figure S3a,b,d). The addition of PEG to the solution decreased all photosynthetic parameters compared to the control for all genotypes except for Ci in Tadmor in the pH 6.0+PEG treatment. Al stress dramatically decreased photosynthetic parameters in all genotypes compared to the control. However, Pn and Gs in XZ29 exhibited the largest increase, and the magnitude of the increase was in the order of XZ29 > XZ5 > Dayton > Tadmor under Al stress relative to the control. The pH 4.0+Al+PEG treatment significantly decreased photosynthetic parameters compared to only Al alone except for Ci in XZ29 (Figure S3a–d).

### 2.3. Activity of H^+^-ATPase, Ca^2+^Mg^2+^-ATPase, and Na^+^K^+^-ATPase in Barley Leaves

**Figure 1:**
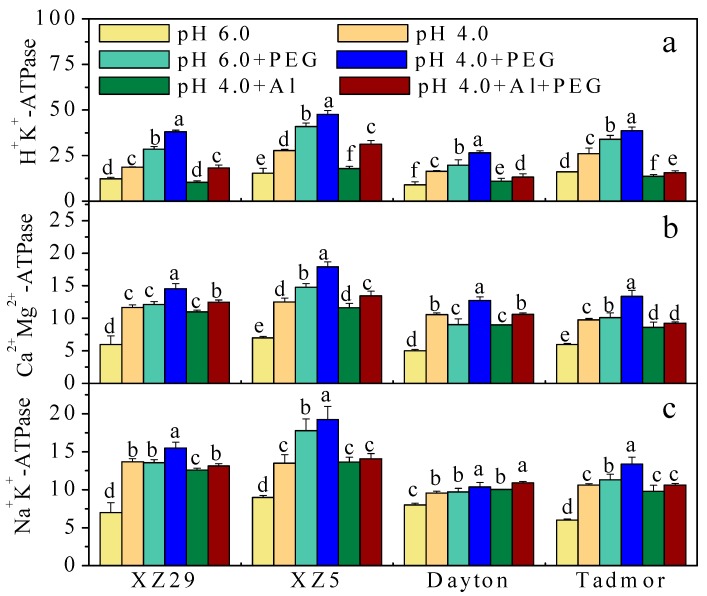
Effects of pH, polyethyleneglycol (PEG) alone, Al alone, and combined (pH 4.0+Al+PEG) stresses on (**a**) H^+^-ATPase, (**b**) Ca^2+^Mg^2+^-ATPase and (**c**) Na^+^K^+^-ATPase of four barley genotypes seven days after treatment. Error bars represent SD values (*n* = 6). Different letters indicate significant differences among the treatments within each genotype according to Duncan’s multiple range tests with *p* < 0.05.

The effects of different stress treatments on H^+^-ATPase, Ca^2+^Mg^2+^-ATPase, and Na^+^K^+^-ATPase of barley leaves are presented in Figure 1. The activity of H^+^-ATPase was significantly increased relative to the control in all genotypes when plants were treated with pH and PEG. In contrast, the activity of this enzyme was significantly decreased in all genotypes under Al stress alone compared to those in the pH 4.0 treatment without Al and PEG. The pH 4.0+Al+PEG treatment significantly reduced H^+^-ATPase relative to PEG alone but increased it compared to Al stress alone. Among the tested three genotypes, XZ5 showed the greatest increase in H^+^-ATPase in response to PEG alone and combined stresses of PEG and Al (Figure 1a). Similar trends were observed in the case of Ca^2+^Mg^2+^-ATPase and Na^+^K^+^-ATPase (Figure 1b,c). The average percent increases in activities of Ca^2+^Mg^2+^ - and Na^+^K^+^-ATPases among the four genotypes in descending order were as follows: XZ5 > XZ29 > Tadmor > Dayton under all stress treatments.

### 2.4. H_2_O_2_, O_2_^−^, and Malondialdehyde (MDA) Contents and Lipoxygenase (LOX) Activity

**Figure 2:**
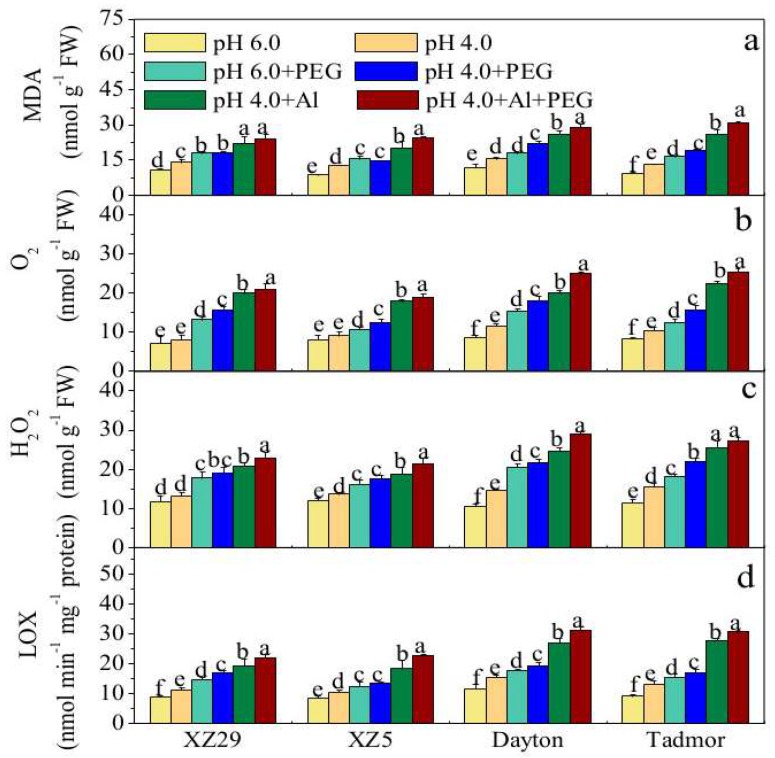
Effects of pH, PEG, Al, and pH 4.0+Al+PEG stresses on (**a**) H_2_O_2_, (**b**) O_2_^−^, (**c**) Malondialdehyde (MDA), and (**d**) Lipoxygenase (LOX) activity of four barley genotypes seven days after treatment. Error bars represent SD values (*n* = 6). Different letters indicate significant differences among the treatments within each genotype according to Duncan’s multiple range test with *p* < 0.05.

The H_2_O_2_, O_2_^−^, and Malondialdehyde (MDA) contents and Lipoxygenase (LOX) activity in barley leaves are shown in Figure 2. A substantial increase in H_2_O_2_, O_2_^−^, and MDA contents and LOX activity was observed in all genotypes under the low pH 4.0 stress relative to the pH 6.0 treatment (Figure 2a–d). The addition of PEG to the growth medium further increased the H_2_O_2_, O_2_^−^, and MDA content and LOX activity in leaves of all barley genotypes. When plants were exposed to Al stress, the H_2_O_2_, O_2_^−^, and MDA content and LOX activity in the leaves of barley plants significantly increased. However, the H_2_O_2_, O_2_^−^,and MDA contents and LOX activity further increased when plants were exposed to pH 4.0+Al+PEG relative to these treatments applied singly. Among the four genotypes, the average percent increases in activities of H_2_O_2_, O_2_^−^,and MDA contents and LOX activity in descending order were as follows: XZ5 > XZ29 > Tadmor > Dayton under all stress treatments (Figure 2a–d).

### 2.5. Antioxidant Enzyme Activities

**Figure 3:**
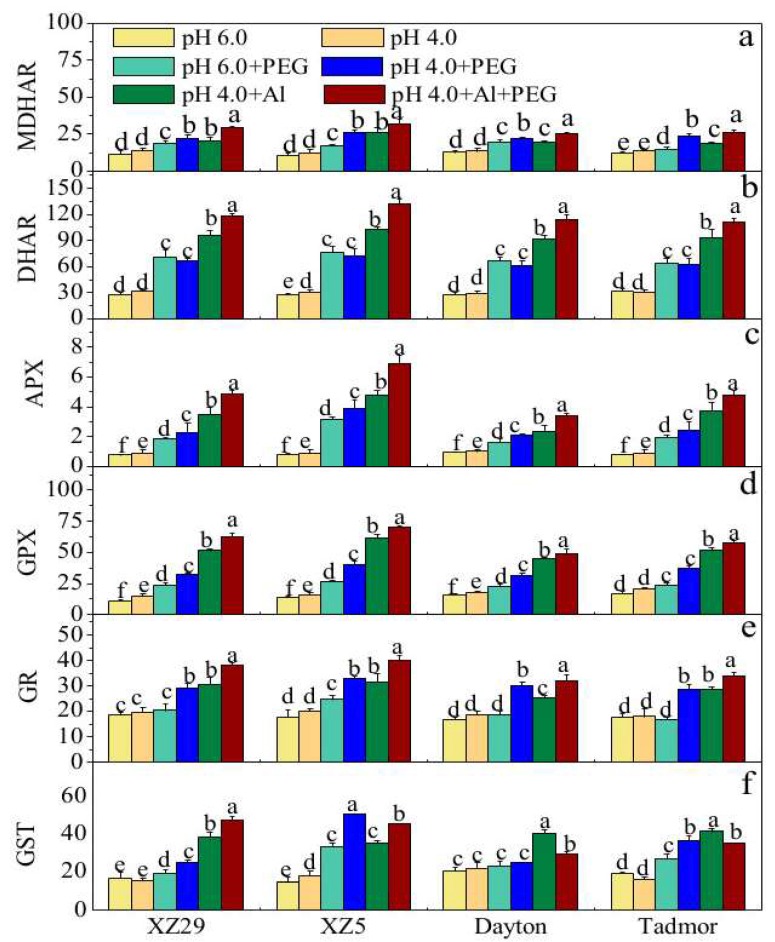
Effects of pH, PEG, Al, and pH 4.0+Al+PEG stresses on (**a**) monodehydroascorbate reductase (MDHAR), (**b**) dehydroascorbate reductase (DHAR), (**c**) ascorbate peroxidase (APX), (**d**) glutathione peroxidase (GPX), (**e**) glutathione reductase (GR), and (**f**) GSH S-transferase (GST) of four barley genotypes seven days after treatment. Error bars represent SD values (*n* = 6). Different letters indicate significant differences among the treatments within each genotype according to Duncan’s multiple range test with *p* < 0.05.

The effects of the pH, Al, and PEG treatments on the activities of the antioxidant enzymes, monodehydroascorbate reductase (MDHAR), dehydroascorbate reductase (DHAR), ascorbate peroxidase (APX), glutathione peroxidase (GPX), GSH S-transferase (GST),and glutathione reductase (GR), are shown in Figure 3a–f. For plants exposed to low pH 4.0, no significant difference was found among the genotypes, except for APX and GPX. However, the addition of PEG and Al to the solution increased the activities of these enzymes compared to the control. XZ5 exhibited the largest increase, and the magnitude of the increase was in the order of XZ5 > XZ29 > Tadmor > Dayton. When the plants were exposed to pH 4.0+Al+PEG, enzyme activities were further increased relative to the single stress treatments in the order of XZ5 > XZ29 > Tadmor > Dayton, expect for GST in Tadmor and Dayton, which was reduced relative to Al stress alone (Figure 3a–f).

### 2.6. Contents of Non-Enzymatic Antioxidants

**Figure 4:**
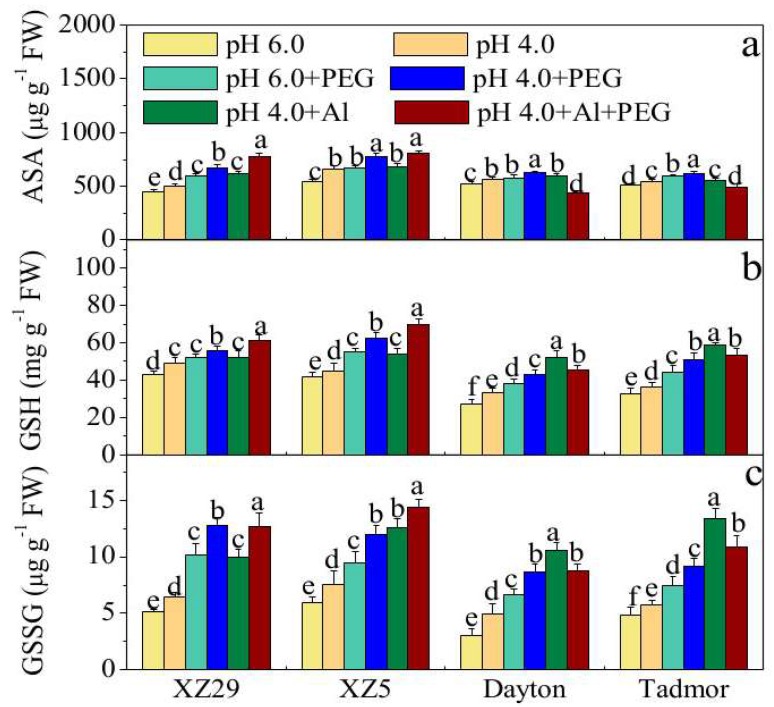
Effects of pH, PEG, Al, and pH 4.0+Al+PEG stresses on the levels of non-enzymatic antioxidants (**a**) reduced ascorbate (ASA), (**b**) reduced glutathione (GSH), and (**c**) oxidized glutathione (GSSG) of four barley genotypes seven days after treatment. Error bars represent SD values (*n* = 6). Different letters indicate significant differences among the treatments within each genotype according to Duncan’s multiple range test with *p* < 0.05.

The contents of reduced ascorbate (ASA), reduced glutathione (GSH), and oxidized glutathione (GSSG) in plants treated with low pH, PEG, and Al alone or in combination are shown in Figure 4. Low pH resulted in noticeable changes to the contents of these antioxidants in all genotypes (Figure 4a–c). Treatment with Al caused an increase in the contents of these antioxidants in the leaves of all four genotypes. The effect of PEG stress on antioxidant contents varied with pH and genotype. The combined treatment of PEG and Al resulted in further increases in antioxidant contents relative to a single stress in Tibetan wild barley but, interestingly, decreased them in cultivated barley relative to Al stress alone (Figure 4a–c). ASA, GSH, and GSSG contents increased more in the two Tibetan wild barley genotypes (XZ29 and XZ5) than in the cultivated barley genotypes (Tadmor and Dayton). The contents of these antioxidants also showed an increase in leaves under pH 4.0+Al+PEG over the controls.

### 2.7. Methylglyoxal (MG) Content and Activity of Glyoxalase (Gly) I and Gly II Enzymes

**Figure 5:**
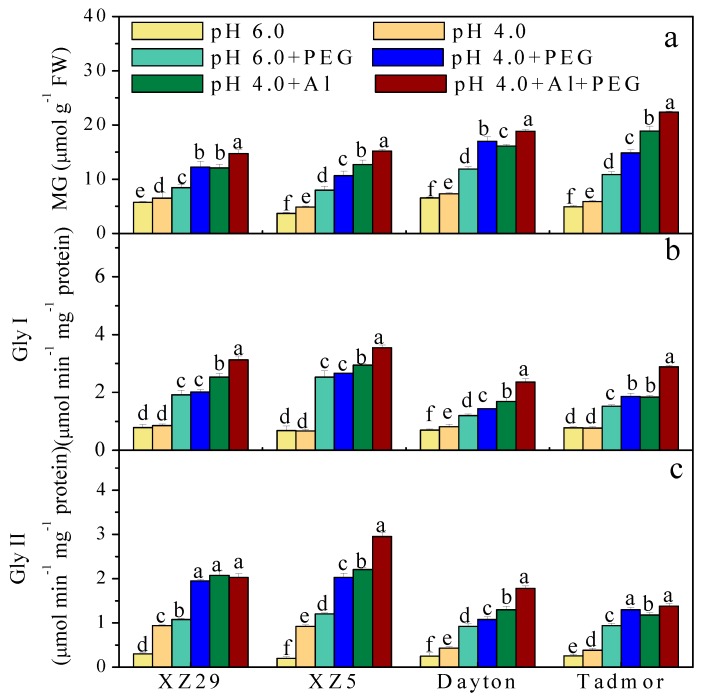
Effects of pH, PEG, Al, and pH 4.0+Al+PEG stresses on the (**a**) MG content and (**b**) Gly I and (**c**) Gly II activities of four barley genotypes seven days after treatment. Error bars represent SD values (*n* = 6). Different letters indicate significant differences among the treatments within each genotype according to Duncan’s multiple range test with *p* < 0.05.

The effects of different treatments on MG content and the activities of Gly I and Gly II enzymes in barley leaves are presented in Figure 5. When plants were exposed to low pH, the MG content significantly increased compared to the control (pH 6.0). Significant increases in MG were observed in all genotypes under PEG stress. Al stress alone significantly increased the MG content in all genotypes compared to pH 4.0 treatments without Al and PEG (Figure 5a). Combined Al and PEG treatment significantly increased the MG content compared to PEG and Al stresses alone. Compared to the Tibetan wild genotypes, Dayton and Tadmor had larger increases in MG content under PEG, Al alone, and pH 4.0+Al+PEG stresses (Figure 5a). Moreover, the activities of the MG detoxifying enzymes Gly I and Gly II in all four genotypes were increased significantly under Al and PEG treatments alone and under the combined Al and PEG treatment, and those of Gly II increased at low pH when compared to the control. Among the four genotypes, the average increases over all treatments in Gly I and Gly II were in the order of XZ5 > XZ29 > Tadmor > Dayton (Figure 5b,c).

### 2.8. DNA Damage Induced by Drought and Al Stress

**Figure 6:**
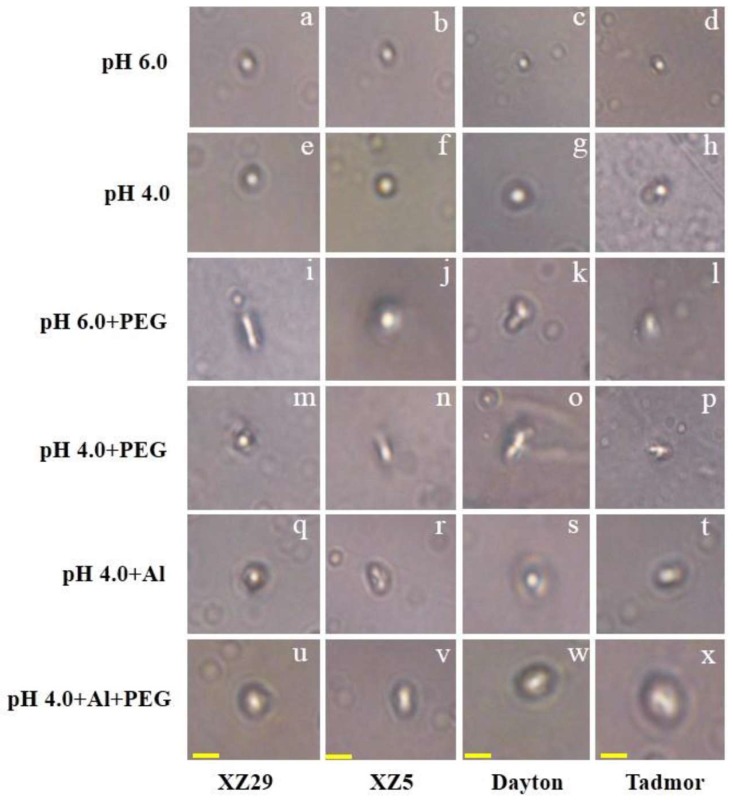
DNA damage analyses of leaves in of Tibetan wild barley XZ29 (left panel), XZ5 (left middle) and cv. Dayton (right middle), Tadmor (right panel) grown under pH 6.0 (a, b, c, d), pH 4.0 (e, f, g, h), pH 6.0+PEG (i, j, k, l), pH 4.0+PEG (m, n, o, p), pH 4.0+Al (q, r, s, t) and pH 4.0+Al+PEG (u, v, w, x) stresses, respectively, for seven days. Images were taken using a fluorescence microscope (BX50WI; Olympus, Shinjuku, Tokyo, Japan) equipped with a digital charge-coupled device (CCD) camera (Olympus). Bar = 40 μm.

The comet assay, being a sensitive technique for the evaluation of DNA damage, was used to evaluate genotypic differences between Al and drought stresses alone or in combination (Figure 6). Under control conditions, no DNA damage was observed in any genotype, and the DNA appeared as a densely condensed structure resembling a bead (Figure 6a–d), and a similar result was found for those plants exposed to low pH (Figure 6e–h). However, barley plants treated with PEG, Al, and pH 4.0+Al+PEG showed a treatment-dependent but genotype-independent increase in leaf DNA damage. Under the pH 4.0 + PEG, pH 4.0 + Al, and pH 4.0 + Al + PEG treatments, larger DNA damage was observed compared to the other treatments (Figure 6i–x). Among the four genotypes, XZ29 and XZ5 (Tibetan wild barleys) had less DNA damage compared to Tadmor and Dayton (cultivated barleys).

### 2.9. Ultrastructural Changes in Chloroplast

**Figure 7:**
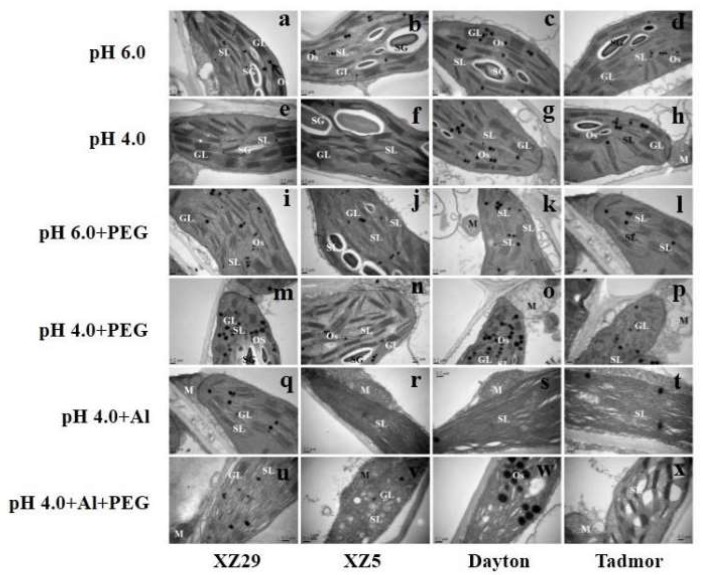
Transmission electron micrograph of chloroplasts of Tibetan wild barley XZ29 (left panel), XZ5 (left middle) and cv. Dayton (right middle), Tadmor (right panel) grown under pH 6.0 (a, b, c, d), pH 4.0 (e, f, g, h), pH 6.0+PEG (i, j, k, l), pH 4.0+PEG (m, n, o, p), pH 4.0+Al (q, r, s, t) and pH 4.0+Al+PEG (u, v, w, x) stresses respectively, for seven days. All individual figures scale bar = 0.2 μm. GL, granum lamellae; M, mitochondrion; Os, osmophilic plastoglobuli; SG; starch grain; SL, stroma lamellae; CW, cell wall.

The chloroplasts in all genotypes were normal under control conditions, but under stress, injuries became apparent in all genotypes (Figure 7). Chloroplasts in control leaves contained dense thylakoids arranged in grana and membranes interconnecting with grana and their stroma lamellae also had localized starch grains (Figure 7a–d). Plants of all four genotypes exposed to low pH did not show noticeable damage to the chloroplasts in all genotypes (Figure 7e–h). Under PEG stress, there was a visible rearrangement of thylakoid membranes and reduced starch grains in the chloroplasts. These changes led to an almost complete unstacking of grana and a loose structure of thylakoids in all genotypes (Figure 7i–l). In the pH 4.0+PEG treatment, the chloroplasts contained a larger number of plastoglobulins compared to those treated with pH 6.0+PEG, especially in XZ29 and Dayton (Figure 7m–p). Al-treated plants had comparatively more changes in chloroplast ultrastructure compared to the PEG-treated plants. In particular, the number of granal stacks was considerably reduced, the thylakoids were loosely arranged, and the thylakoid network was visibly incomplete (Figure 7q–t). Under pH 4.0+Al+PEG, significant changes were observed: the chloroplasts were swollen, their thylakoid membranes showed dilations, the spaces between the membranes looked swollen, and undulated thylakoid areas developed (Figure 7u–x). Among the four genotypes, XZ29 and XZ5 showed less change than did Tadmor and Dayton subjected to pH 4.0+Al+PEG (Figure 7u–x).

## 3. Discussion

### 3.1. The Reduction of Gas Exchange Associated with Combined Stress

Drought and Al stress cause a significant reduction in the photosynthetic rate in different plant species [23,24] In the authors’experiment, barley plants showed a rapid decrease in Gs, Pn, and Tr when grown under PEG, Al alone, and combined pH 4.0+Al+PEG stress. In this work, decreased Pn was associated with significantly reduced Gs and Tr, showing that stomatal factors inhibit photosynthesis. A substantial reduction in Gs could have resulted from the closure of stomata due to stress induction by the pH4.0+PEG+Al treatment; this reduction is an important stress avoidance mechanism in plants. Moreover, Al and PEG caused a reduction in photosynthetic parameters (Figure S3) and reduced plant growth and biomass (Figures S1 and S2), suggesting that Al in combination with PEG disrupted chloroplast and Chl-protein structures and deactivated the enzymes of the Calvin cycle, thereby impairing photosynthesis in the genotypes [25].Thus, plants can avoid stresses by reducing transpiration.

### 3.2. ATPase Activity is Critical for Drought and Al Tolerance

This study showed that barley plants subjected to pH 4.0+PEG+Al combined stress were necrotic compared with those under drought and Al stresses alone. Tibetan wild barley XZ5 and XZ29 had higher tolerance to combined stresses of pH 4.0+PEG+Al, with less reduction in plant height, biomass, and Pn, and a lower Al content, relative to Dayton and Tadmor.

The key function of H^+^-ATPase is to generate an electrochemical proton (H^+^) gradient, driving ion and metabolite transport across the cell membrane via secondary transport systems [26]. In this study, both XZ29 and XZ5 maintained significantly higher H^+^-ATPase, Ca^2+^Mg^2+^-ATPase, and Na^+^K^+^-ATPase activities under stress than did Dayton and Tadmor (Figure 2a–c), suggesting that ATPase activity is associated with drought and Al tolerance in Tibetan barley. This is supported by the authors’ previous studies that showed an increase in Na^+^K^+^-ATPase activity in barley plants subjected to drought and salinity stresses [27]. In addition, the higher Ca^2+^Mg^2+^-ATPase activity could partly explain the fewer symptoms triggered by Al toxicity in XZ29 and XZ5, since Al directly competes with Ca^2+^ and Mg^2+^ binding sites associated with entry of the minerals into cells, thereby reducing Ca^2+^ and Mg^2+^ influx [28]. These results illustrated that Tibetan wild barley under combined stresses of low pH, drought, and Al increased H^+^-ATPase and Ca^2+^Mg^2+^-ATPase activities in the leaf plasma membrane, and this is associated with Al tolerance in barley. However, the dynamic changes of Al-modulated Ca^2+^Mg^2+^-ATPase in plasma membrane, vacuolar, and other cell membranes of cultivated and Tibetan wild barley remain ideal targets for further investigations in the future.

### 3.3. Tibetan Wild Barley Exhibited Higher ROS-Detoxifying Capacity under Combined Stress Conditions

Many kinds of environmental stresses have been shown to increase the levels of ROS in plant cells, including those associated with Al, drought, and salinity. ROS-detoxifying mechanisms are inherent in plants. Along with increased Al accumulation, this study recorded augmented levels of O_2_^−^ and H_2_O_2_ (Figure 2a,b) in the seedlings of Tibetan barley that might be attributed to oxidative bursts caused by drought and Al stresses in seedlings. Increased ROS and MDA levels induced by PEG and Al (Figure 2) suggested that PEG and Al stresses inflicted severe damage to the membranes of the barley seedlings. This membrane lipid peroxidation was attributed to an increase in the activity of LOX (Figure 2). This finding is in line with previous results, that is, Al induces lipid peroxidation and ROS accumulation in tea leaves [2].

The essential role of antioxidative systems for maintaining a balance between the overproduction of ROS and scavenging them to keep them at appropriate levels for signaling and reinstatement of metabolic homeostasis is well established [29]. This study showed that wild barley regulates the level of H_2_O_2_ by enhancing the activities of APX and DHAR, while maintaining the activities of MDHAR and GR above control levels (Figure 2 and Figure 3). Plants can induce antioxidant enzymes, including GPX, APX, GST, and GR, to counteract oxidative damage. GR is involved in maintaining a high ratio of GSH/GSSG, which is required for the regeneration of ascorbate (AsA), an important antioxidant in plant cells [30]. The authors’ findings indicate that imbalanced GSSG recycling as influenced by GR might have induced the depletion of GSH and GSSG under the pH 4.0+Al+PEG treatment (Figure 4), which affected GST activity as a consequence (Figure 3). Under the pH 4.0+Al+PEG treatment, XZ5 and XZ29 had higher levels of ASA and GSH compared to Dayton and Tadmor (Figure 4); these compounds would scavenge ROS and give improved protection against oxidative damage induced by the stresses. Therefore, it is conceivable that XZ5 and XZ29 exhibited higher relative tolerance of the combined stresses of drought and salinity by acquiring more effective antioxidant mechanisms to overcome damage arising from ROS accumulation.

### 3.4. XZ29 and XZ5 Maintained a Higher Increase in Activities of Gly I and Gly II with Lower MG Toxicity and DNA Damage under Stress

Plants under abiotic stresses over produce MG, which consequently leads to cellular toxicity and oxidative damage [31,32]. In this study, as shown in Figure 5, the relatively lower MG content accumulated due to drought and Al stresses in XZ29 and XZ5 could be attributed to greater activities of GlyI and Gly II (Figure 5), indicating that wild barley is a potential source of alleles for PEG/Al stress tolerance. The relatively lower activities of Gly I and Gly II might exacerbate the toxicity of Al in the cultivated barley. This result also suggests that the increased activity of Gly II enhanced the recovery of GSH and recycled it into the system. The results of this study imply that ROS and MG toxicities in Tibetan wild barley induced by Al and drought are mitigated by the biochemical actions of the antioxidant defense and Gly systems. As suggested by several reports, antioxidant defense is closely linked with Gly systems and both work at the same time to confer tolerance to plants subjected to multiple stresses, including heavy metals [33,34]. Therefore, the role of methylglyoxal and glyoxalases (such as Gly I and Gly II) in stress tolerance in barley leaves requires further investigation.

Different types of damage and repair of DNA can be efficiently measured by comet assays, a technique which was detailed by Östling and Johanson [35]. The over-production of ROS due to combined PEG and Al stresses degrades DNA in the comet head more than is caused by either stress alone (Figure 6). In this study, under drought and pH 4.0+PEG+Al, the oxidative and DNA damage was higher in Dayton and Tadmor, whereas less damage was found in XZ5 and XZ29, indicating the potential removal of ROS in XZ5 and XZ29. This kind of DNA damage was also reported by Ahmed et al. in barley exposed to drought and salinity stress [36].

### 3.5. Tibetan Wild Barley Showed Fewer Changes in Chloroplasts Organelles under Combined Stress

Chloroplasts, mitochondria, and plasma membranes of plant cells are major sites of ROS generation under stress conditions [37]. In this study, ultrastructural investigation revealed that PEG, Al alone, and combined (pH 4.0+PEG+Al) stresses damaged the ultrastructure of chloroplasts, as manifested by the disturbed shape, the dilation of thylakoid membranes, and the accumulation of osmophilic plastoglobuli (Figure 7), with Dayton and Tadmor being greatly affected. These ultrastructural alterations suggest that these stresses induced important disturbances in metabolic functions and the lipid composition of chloroplast membranes. Moreover, numerous large plastoglobuli appeared, accompanied by the disruption of the chloroplast membrane system due to PEG treatment (Figure 7), particularly in XZ29 and Dayton, confirming the results of Pääkkönen et al. [38]. Genotypic differences in the ultrastructural damage could partially account for the genetic differences in Pn reduction and ROS accumulation. The authors’ investigation also revealed that Al obviously damaged the ultrastructure of chloroplasts, with the effects being most severe in Tadmor (Figure 7). Rauser and Samarakoon reported that Ni, Co, and Zn induce starch accumulation, probably through the inhibition of vein loading [39]. However, in this experiment, the authors observed that Al stress reduced the starch accumulation in chloroplast stroma, with the changes in Tadmor being much more obvious.

## 4. Materials and Methods

### 4.1. Plant Materials and Hydroponic Culture

A greenhouse hydroponic experiment was carried out in Zhejiang University in Hangzhou, China using two Tibetan wild barley lines (XZ5, drought-tolerant; XZ29, Al-tolerant) and two cultivated barley lines (Al-tolerant Dayton; drought-tolerant Tadmor) as detailed in the authors’ previous study [22]. Twelve days after transplanting, 100 μmol/L Al (as AlCl_3_·6H_2_O) and/or 20% (*w*/*v*) PEG (M_r_6000) [40] were added to the culture solution to form the following six treatments: pH 6.0 (control, basal nutrient solution (BNS) at pH 6.0); pH 4.0 (BNS at pH 4.0); pH 6.0+PEG (BNS+20% PEG, pH 6.0); pH 4.0+PEG (BNS+20% PEG, pH 4.0); pH 4.0+Al (BNS+100 μmol/L Al, pH 4.0); and pH 4.0+PEG+Al (BNS+20% PEG+100 μmol/L Al, pH 4.0). A split-plot design was adopted for the experiment where each combination was replicated six times, and each replication contained fourteen plants.

Plants were harvested after seven days of treatment, and leaf samples were collected to measure DNA damage, ultrastructure, morphological (plant height and dry weight), physiological (photosynthetic parameters, ATPase, and Al concentration) and biochemical parameters, namely, reduced glutathione (GSH), reduced ascorbate (ASA), lipid peroxidation, hydrogen peroxide (H_2_O_2_) and superoxide radical (O_2_^−^), lipoxygenase (LOX), monodehydroascorbate reductase (MDHAR), dehydroascorbate reductase (DHAR), GSH *S*-transferase, methylglyoxal (MG), glyoxalase (Gly I) and Gly II activities, and enzymatic antioxidants. Deionized water was used for preparing stock solutions with reagents with analytical grade.

### 4.2. Measurement of Plant Height, Dry Weight, and Al Concentration

Plants were gently uprooted and their height was measured. The weights of plant samples were taken after drying at 80 °C for 24 h. After being ground, the shoots were digested with HNO_3_:HClO_4_ (4:1, *v*/*v*) at 150 °C for 6 h, and inductively coupled plasma-atomic emission spectrometry (ICP/AES) (IRIS/AP optical emission spectrometer, Thermo Jarrell Ash, San Jose, CA, USA) was used for the analysis of the Al concentrations in the shoots.

### 4.3. Determination of Photosynthetic Parameters

An LI-COR photosynthetic system (model LI-6400, LI-COR Inc., Lincoln, NE, USA) was used to determine transpiration rate (Tr), net photosynthetic rate (Pn), intercellular CO_2_ concentration (Ci), and stomatal conductance (Gs) on the second fully expanded leaves after seven days of treatment.

### 4.4. Determination of ATPase and Non-Enzymatic Antioxidants

ATPase and reduced glutathione (GSH) activities in leaves were determined using a kit (Jiancheng Bio Co., Nanjing, China) (www.njjcbio.com/html/search.php) following the manufacturer’s instructions. Assays of ASA content were performed spectrophotometrically at 265 nm in line with Dutilleul et al. [41]. Oxidized glutathione or glutathione disulfide (GSSG) was determined according to Griffiths based on enzymatic recycling [42].

### 4.5. Determination of Lipid Peroxidation, Hydrogen Peroxide (H_2_O_2_), and the Superoxide Radical (O_2_^−^)

Malondialdehyde (MDA) was measured to determine the level of lipid peroxidation following the method used by Ahmed et al. at 532 nm and assuming an extinction coefficient of 155 mM^−1^·cm^−1^ [43]. The details of determination of H_2_O_2_ and O_2_^−^ extracted from leaves can be found in Ahmed et al. who measured H_2_O_2_ and O_2_^−^ in leaves using a spectrophotometer [22].

### 4.6. Extraction and Assay of Enzymes and Determination of Methylglyoxal (MG) Content

Approximately 0.3 g aliquots of leaf tissue were used for enzyme assays. All procedures were performed at 0–4 °C. Lipoxygenase (LOX, EC1.13.11.12), monodehydroascorbate reductase (MDHAR, EC1.6.5.4), dehydroascorbate reductase (DHAR, EC 1.8.5.1), GSH S-transferase (GST, EC 2.5.1.18), glyoxalase (Gly I, EC 4.4.1.5) and Gly II (EC 3.1.2.6) activities and MG content were measured as described by Mostofa et al. [44]. The methods for determining the activities of glutathione reductase (GR, EC 1.6.4.2), ascorbate peroxidase (APX, EC 1.11.1.11), and glutathione peroxidase (GPX, EC 1.11.1.9) were the same as the methods used by Ahmed et al. [43]. The extracts were also analyzed for total protein content with standard bovine serum albumin (BSA) following the dye-binding method used by Bradford [45].

### 4.7. Single Cell Gel Electrophoresis Assay (Comet Assay)

Comet assays were performed on the barley seedlings following the method used by Ahmed et al. [36]. Images of ethidium bromide-stained comets were captured using a fluorescence microscope (BX50WI; Olympus, Shinjuku, Tokyo, Japan) equipped with a digital charge-coupled device (CCD) camera (Olympus, Shinjuku, Tokyo, Japan).

### 4.8. Investigation of Leaf Ultrastructure

Leaf ultrastructure was also examined seven days after the treatments commenced according to the method used by Cai et al. [46]. Fresh leaves (1 mm^2^, top middle section of the first fully expanded leaf) were fixed in 0.1 M sodium phosphate buffer (PBS, pH 7.4) containing 2.5% glutaraldehyde (*v*/*v*) at room temperature overnight and then washed three times in PBS solution. The washed samples were post-fixed in 1.0% osmium (VIII) oxide for 1 h and then washed three times (10 min each wash) in 0.1 M PBS (pH 7.4). The samples were then dehydrated in an ethanol series 50%, 60%, 70%, 80%, 90%, 95%, and 100% (15–20 min in each solution). Finally, the samples were dehydrated for 20 min in absolute acetone. The samples were infiltrated and embedded in Spurr’s resin overnight. Ultra-thin sections of 80 nm were prepared by heating the specimens at 70 °C for 9 h and then were mounted on copper grids and viewed with a transmission electron microscope (JEOL TEM-1230EX, JEOL Ltd. Japan) using an accelerating voltage of 60.0 kV.

### 4.9. Statistical Analysis

The analysis of samples for physiological traits used six replicates. Statistical analyses were performed using Data Processing System (DPS) software and analysis of variance (ANOVA) followed by Duncan’s Multiple Range Test (DMRT); the treatment effects were evaluated at <5% level of significance. The graphs in the manuscript were prepared by plotting the relevant data in Origin Pro version 8.0 (Origin Lab Corporation, Wellesley Hills, Wellesley, MA, USA).

## 5. Conclusions

**Figure 8:**
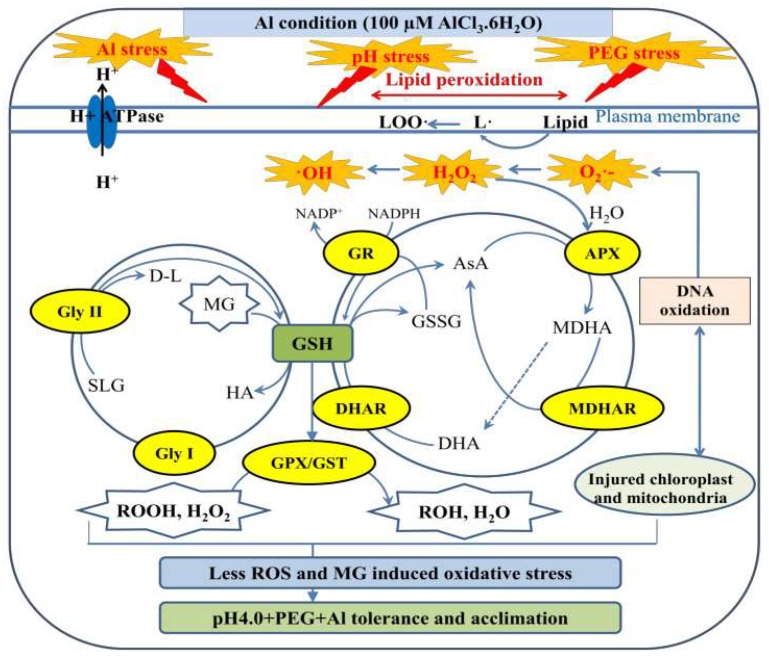
Graphic depiction based on this study’s findings. The potential regulatory mechanisms involved in acclimation to PEG and Al of Tibetan wild barley and how Al and PEG stress interferes with this acclimation are shown. Tibetan wild barley had increased GSH concentrations and showedless damage to chloroplasts, mitochondria, and DNA after treatment with pH 4.0+Al+PEG, which might occur through the induction of ROS production and MG detoxification by increasing Gly I and Gly II activities as well as redox homeostasis, leading to better stress tolerance. The dotted arrow indicates spontaneous conversion. Small and large circles indicate the Gly system and ASA-GSH system, respectively. Arrows indicate the potential connections and dotted arrow indicates spontaneous conversion. AsA, ascorbic acid; APX, ascorbate peroxidase; DHA, dehydroascobate; DHAR, dehydroascorbate reductase; d-l, d-lactic acid; GSSG, oxidized GSH; Gly, glyoxalase; GR, GSH reductase; GPX, GSH peroxidase; GST, GSH S-transferase; HA, hemithioacetals; H_2_O_2_, hydrogen peroxide; MDHAR, monodehydroascorbate reductase; NADPH, nicotinamide adenine dinucleotide phosphate; O_2_^•−^, superoxide; ^•^OH, hydroxyl ion; ROOH, lipid hydroperoxide; SLG, *S*-d-lactoylglutathione.

Based on the above observations, a model depicting the physiological and biochemical mechanisms involving low pH, PEG-induced drought and Al stress tolerance in barley plants is proposed (Figure 8). A number of reactions involved with the ascorbate–glutathione cycle act jointly with H_2_O_2_ metabolism. The recycled GSH prevents oxidative stress by facilitating the homeostasis of GSH and enhanced activities of DHAR, GPX, and GST. Other antioxidative processes (enzymatic and non-enzymatic) also play important roles in a variety of processes involved in plant development and in response to low pH, PEG-induced drought, and Al stress. In barley plants, drought, Al, and low pH protection mediated by antioxidative process might be coupled with less damage to chloroplasts, mitochondria, and DNA. Therefore, it can be suggested that wild barley possesses more effective antioxidant mechanisms than cultivated barley leading to greater stress tolerance (Figure 8). The genetic backgrounds of wild barleys differ from cultivated barleys and they have adapted to cope with harsher environments, such as those affected by drought, salinity, Al, and poor soils, and their germplasm provides hope for crop improvement. However, further studies using advanced molecular techniques and mutant analysis are required to better understand the detailed mechanisms of the response of Tibetan wild barley to combined abiotic stresses.

## Supplementary Materials

Supplementary materials can be found at http://www.mdpi.com/1422-0067/19/11/3553/s1.
