# Supplementary figures and images for: Tolerance to Drought, Low pH and Al Combined Stress in Tibetan Wild Barley Is Associated with Improvement of ATPase and Modulation of Antioxidant Defense System
Source: Int J Mol Sci. 2018 Nov 11;19(11):3553. doi: 10.3390/ijms19113553 (PMC6274725; doi:10.3390/ijms19113553)

Fig. S1.

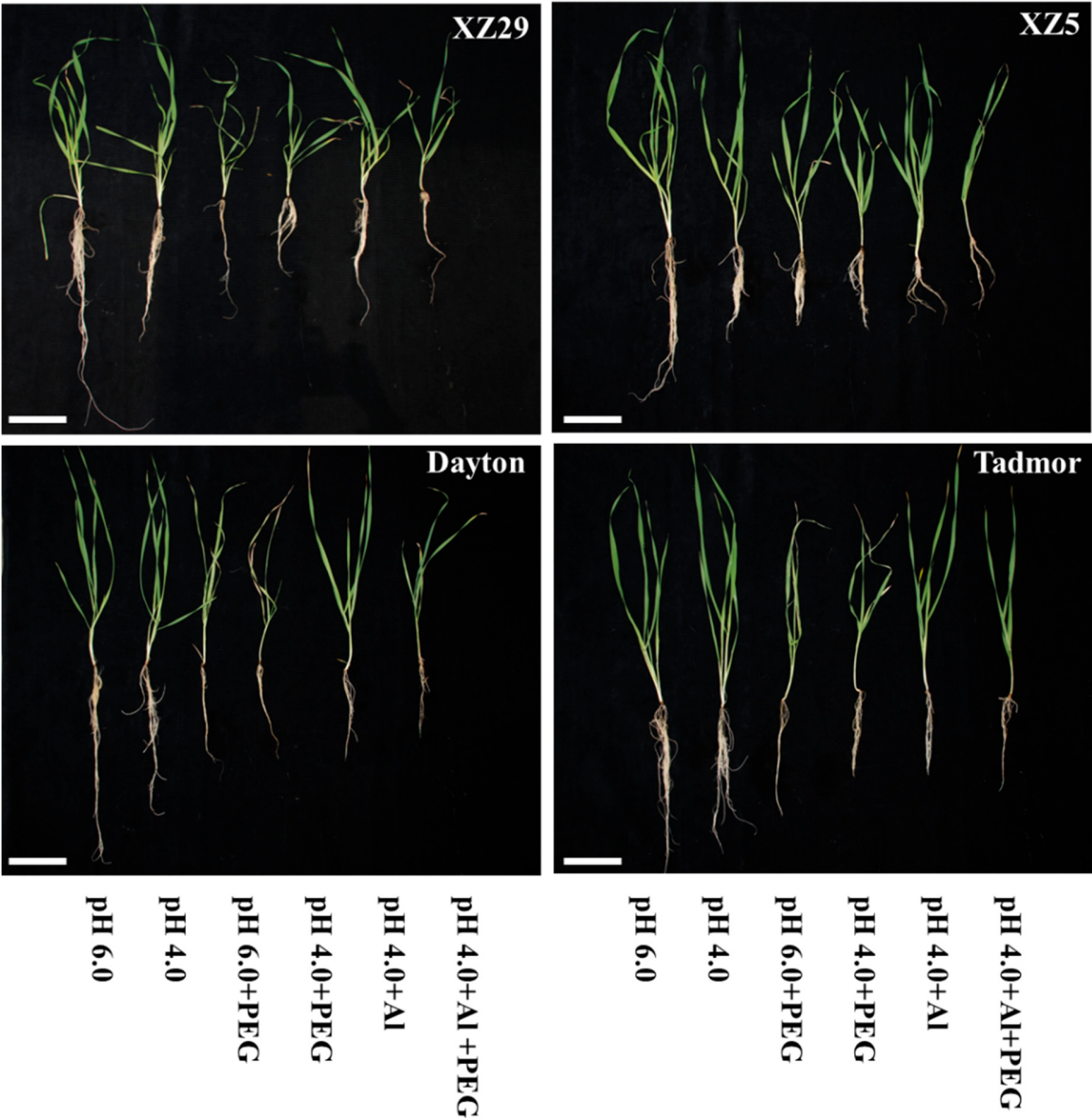

Fig. S2

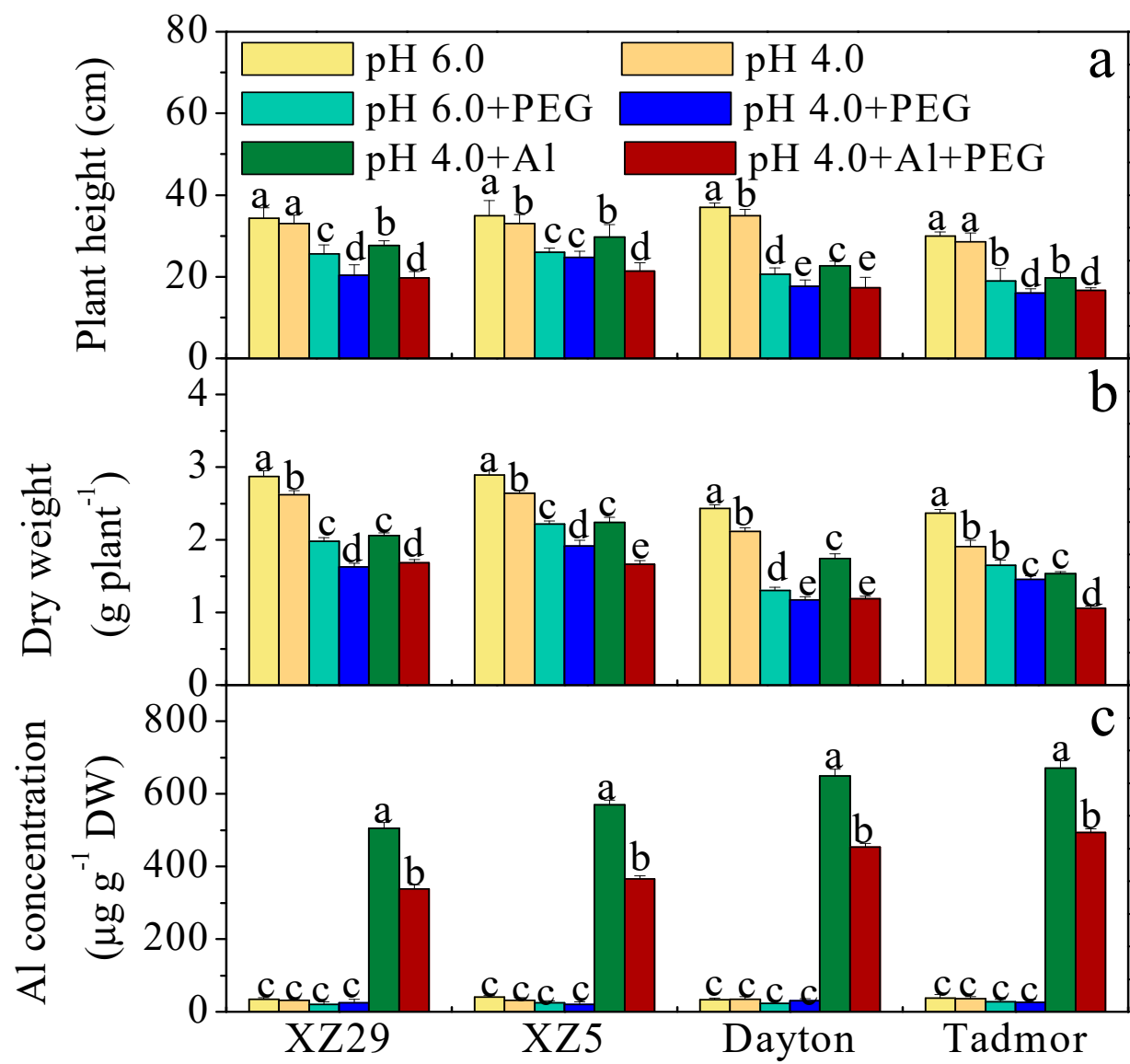

Fig. S3

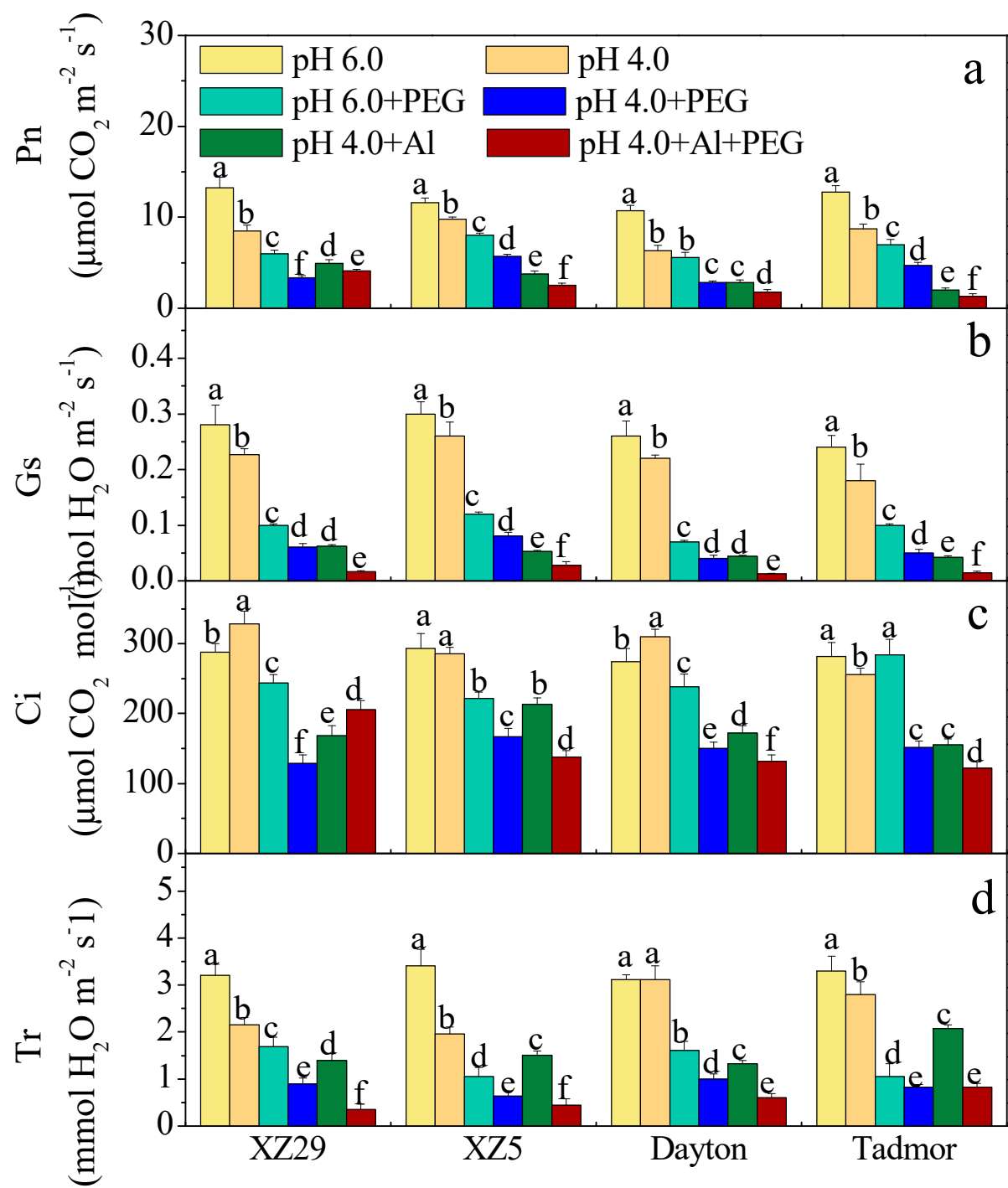

Supplement: Supplementary file 1 [file ijms-19-03553-s001.pdf]
